# Characterization of m6A-related lncRNA signature in neuroblastoma

**DOI:** 10.3389/fped.2022.927885

**Published:** 2022-10-17

**Authors:** Liming Li, Sisi Chen, Jianhong Li, Guochou Rong, Juchao Yang, Yunquan Li

**Affiliations:** Department of Pediatric Surgery, GuiPing People’s Hospital, Guangxi, China

**Keywords:** N6-methyladenosine, prognosis, risk score, neuroblastoma, lncRNAs

## Abstract

N6-methyladenosine (m6A) constitutes one of the most common modifications in mRNA, rRNA, tRNA, microRNA, and long-chain noncoding RNA. The influence of modifications of m6A on the stability of RNA depends upon the expression of methyltransferase (“writer”) and demethylase (“eraser”) and m6A binding protein (“reader”). In this study, we identified a set of m6A-related lncRNA expression profiles in neuroblastoma (NBL) based on the Therapeutically Applicable Research to Generate Effective Treatments (TARGET) program. Thereupon, we identified two subgroups of neuroblastoma (high-risk group and low-risk group) by applying consensus clustering to m6A RNA methylation regulators (“Readers,”, “Writer,” and “Erase”). Relative to the low-risk group, the high-risk group correlates with a poorer prognosis. Moreover, the present study also revealed that the high-risk group proves to be significantly positively enriched in the tumor-related signaling pathways, including the P53 signaling pathway, cell cycle, and DNA repair. This finding indicates that these molecular prognostic markers may also be potentially valuable in early diagnosis, which provides a new research direction for the study of molecular mechanisms underlying the development of NBL. In conclusion, this study constructed a new model of NBL prognosis based on m6a-associated lncRNAs. Ultimately, this model is helpful for stratification of prognosis and development of treatment strategies.

## Introduction

Neuroblastoma (NBL) is a neuronal crest cell derived from the sympathetic nervous system, accounting for 6%–10% of childhood tumors (1). Most children with neuroblastoma exhibit metastatic and/or high-risk characteristics (2). It is moreover responsible for 12% of cancer-related deaths in children under the age of 15 (3). Neuroblastomas feature a wide range of tumor heterogeneity with different clinical manifestations and courses of disease in accordance with tumor biology (4, 5). Patients with medium- and low-risk conditions are mainly treated by operation, and additional chemotherapy can be included if necessary. Advances in the treatment of patients with high-risk diseases include intensive induction chemotherapy and myeloablative chemotherapy followed by differentiation therapy and immunotherapy for minimal residual conditions. These treatments can increase the 5-year overall survival (OS) rate to 50% (6). Due to the fact that the initial symptoms of neuroblastoma are not typical, early accurate diagnosis remains a challenge. Risk stratification therapy helps reduce the intensity of treatment of children with low-and medium-risk conditions, and the establishment of prognostic models helps evaluate the treatment of high-risk children. Therefore, it is of great significance to determine the risk characteristics in the diagnostic stage for the purpose of evaluating the prognosis of children with neuroblastoma.

Epigenetics, involving changes in DNA or histones, has been widely studied in tumor progression (7). As a class of epigenetics, RNA modification has become a key regulator of RNA function and metabolism (8). N6-methyladenosine (m6A) constitutes one of the most common modifications in mRNA, rRNA, tRNA, microRNA, and long-chain noncoding RNA (9, 10). Dynamic m6A modification affects a variety of cellular processes, such as RNA stability, output, splicing, or translation (11). The effect of m6A on the stability of RNA depends on m6A readers, including YTHDF1, YTHDF2, YTHDF3, and YTHDC (12). In addition, m6A modification is regulated by two other enzymes: “Writer” (methyltransferase, including WTAP, KIAA1429, RBM15/15B, and METTL3/14/16) and “Erase” (demethylase, including ALKBH5 and FTO) (13). Increasing evidence proves that m6A-related lncRNAs can serve as a new potential target for prognosis and developing individualized therapies for a variety of cancers (14–16). However, the relationship between m6A methylation and NB remains unclear. Recently, a study reported that CoCl decreased the activity of demethylase and significantly changed the level of mA modification by affecting the expression of mA methyltransferase and demethylase in human neuroblastoma H4cells (17). In addition, it has been reported that miR-98/MYCN axis-mediated inhibition of neuroblastoma progression requires RNA mA modification (18). On top of that, the risk prediction model constructed by five m6A-related genes (*METT14*, *WTAP*, *HNRNPC*, *YTHDF1*, and *IGF2BP2*) is helpful in the clinical prognosis of NBL (19).

In this study, we identified a set of m6A-related genomic expression profiles in NB based on the *Therapeutically Applicable Research to Generate Effective Treatments* (TARGET: https://ocg.cancer.gov/programs/target) program. Subsequently, several lncRNAs related to m6A regulatory factors were identified as potential biomarkers. To evaluate the impact of m6A-related lncRNA on prognostic value, we eventually identified seven m6A-related lncRNAs that enabled us to construct a risk score, after which NB cases were divided into a high-risk group and a low-risk group based on the median risk score. Since then, we have analyzed the prognostic role of the risk score in NBL. Our results suggest that the risk score of m6A-related lncRNA may provide information for risk assessment and prognosis stratification. More importantly, the prognostic correlation of the risk marker was successfully verified in the internal subgroup analysis.

### Data source and processing

**Figure 1:**
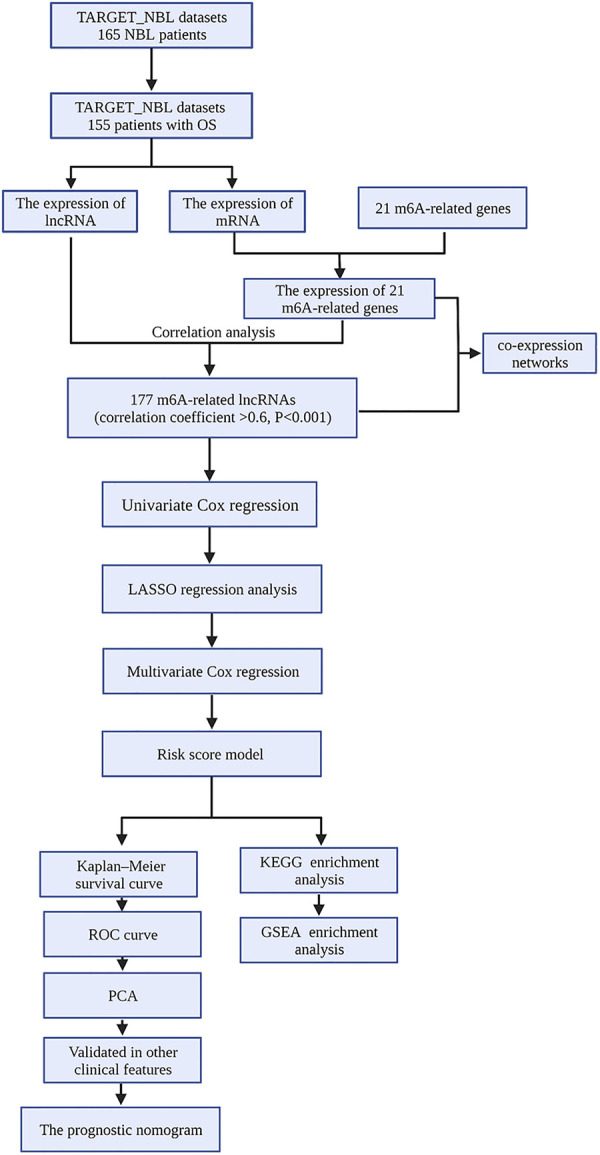
Analysis flow chart.

The fragments per kilobase million (FPKM) data and the corresponding clinical information of NBL samples were downloaded from TARGET Data Matrix (https://ocg.cancer.gov/programs/ target/data-matrix). NBL samples without prognostic information were excluded. In our study, a total of 155 NBL patients with complete OS information were enrolled (Supplementary file S1 and S2). The data were annotated and collapsed into mRNAs and lncRNAs employing Ensembl (http://asia.ensembl.org) and using the Perl program. Finally, the lncRNA and mRNA expression profiles were extracted separately from the database. The flowchart of our work is shown in Figure 1.

### Identification of m6A methyltransferase-related lncRNAs

Twenty-one m6A-related genes were extracted based on previous studies including the Writers (*METTL3, METTL14, RBM15, RBM15B, WTAP, KIAA1429, CBLL1*, and *ZC3H13*), Erasers (*ALKBH5* and *FTO*), and Readers (*YTHDC1, YTHDC2, YTHDF1, YTHDF2, YTHDF3, GF2BP1, HNRNPA2B1, HNRNPC, FMR1, LRPPRC,* and *ELAVL1*) (20, 21). In addition, Pearson correlation analysis was performed in the statistical software R (version 4.0.3) to identify the potential lncRNA associated with m6A-related genes. A total of 177 m6A-related lncRNAs with Pearson correlation coefficients greater than 0.6 and *P* < 0.01 were included in subsequent studies, and subsequently, the coexpression networks between m6A genes and m6A-related lncRNAs were visualized using the Cytoscape software (v3.08).

### Construction of the prognostic signature

By means of univariate Cox regression analysis, m6A-related lncRNAs that can be used to predict the prognosis of patients were screened. In addition, a forest plot was used to visualize the hazard ratio (HR) of these prognostic-related lncRNAs. Subsequently, the LASSO regression method was used to screen the best candidate, and a multifactor Cox regression analysis was carried out to establish the Cox risk assessment model by means of R packages (“survival” and “glmnet”). Afterward, the samples were divided into a high-risk group and a low-risk group based on median risk scores for both sets.

### Evaluation of the risk score model

Based on the median value of the risk score, the heat map, risk curves, and scatter plots were plotted to show the differences in prognosis between the high-risk group and the low-risk group through the R packages (“pheatmap,” “survival,” and “survminer”). The Kaplan–Meier survival curve was used to compare the OS between the two groups. Furthermore, the receiver operating characteristic (ROC) curve and area under curve (AUC) were harnessed to evaluate the diagnostic and prognostic value of the risk score model and clinicopathological features. In turn, we combined the clinical data of patients and the risk model constructed in this study for univariate and multivariate Cox analyses and obtained the related forest plots using a survival R package. To further explore the impact of single-target lncRNA on NBL patients in the prognostic risk model, the Wilcoxon test was employed for a comparison of the relationship between the lncRNA expression level of each target and clinical parameters. Principal component analysis (PCA) was employed in order to perform effective dimension reduction, pattern recognition, and exploratory visualization analysis on the expression profiles of the whole genome, m6A-related genes, m6A-related lncRNAs, and seven risk model lncRNAs.

### Gene set enrichment analysis

Gene set enrichment analysis (GSEA) was performed in the NBL cohort to determine the underlying biological processes and cellular pathways of high-risk and low-risk subsets identified by m6A-associated lncRNA expression characteristics. A set of genes with a false discovery rate <0.25 and a standardized *P* value <0.05 were considered significant.

### Establishment and validation of a prognostic nomogram

Based on risk score and prognostic clinical variables, we constructed a prognostic nomogram to quantitatively predict the prognosis of NBL patients. Subsequently, the concordance index (C-index) and calibration curves were employed to evaluate the reliability and accuracy of the prognostic nomogram. At this juncture, the “rms,” “foreign,” and “survival” packages were employed.

### Statistical analysis

All statistical analyses were performed using R software (version 4.0.3). The programming language Perl (version 5.30.2) was implemented for data processing. The network was visualized using the Cytoscape software (v3.08).

## Results

### Identification of m6A-related lncRNA

**Figure 2:**
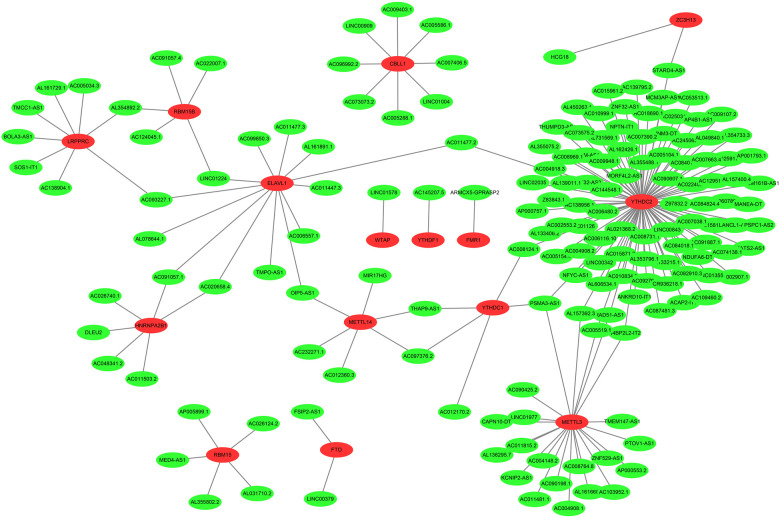
The coexpression networks between m6A genes and m6A-related lncRNAs. 177 lncRNAs co-expressed with m6A-related genes. The red nodes represent m6A genes and green nodes represent m6A-related lncRNAs.

The neuroblastoma-related RNA sequencing data were downloaded from the Target database, which included 155 neuroblastoma tissue samples featuring complete clinical information. Subsequently, the expression profiles of m6A-related genes were obtained from the neuroblastoma-related RNA sequencing data. As a result, we discovered that 177 lncRNAs were significantly associated with M6A-related genes utilizing Pearson correlation analysis (correlation coefficient >0.6, *P* < 0.001), which was visualized by Cytoscape (Figure 2).

### Construction of an MRlncRNA signature

**Figure 3:**
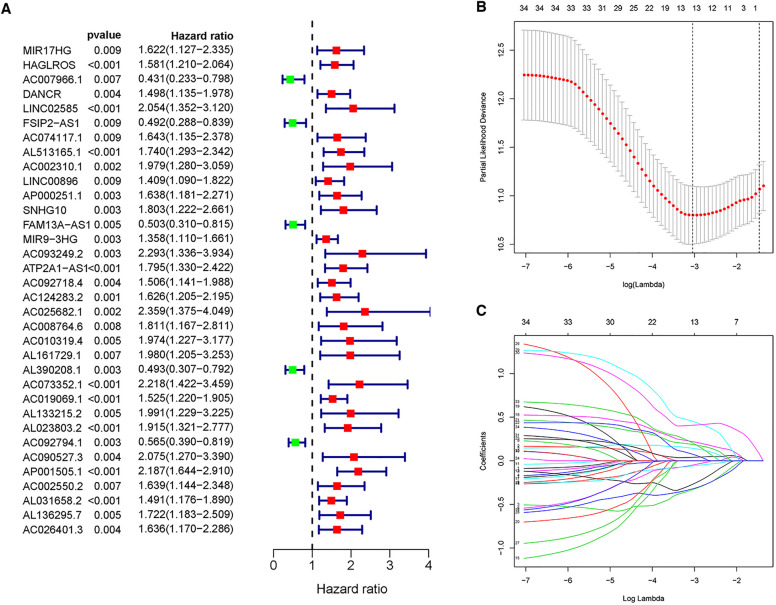
Univariate Cox regression analysis and LASSO regression analysis. (**A**) Forest plot of univariate Cox regression showed that 34 MRlncRNAs were prognostic factors of NBL. (**B,C**) LASSO regression was performed, calculating that the accuracy of the MRlncRNAs-related prediction model was the best when 13 MRlncRNAs were selected.

Univariate Cox regression analysis is capable of providing clues for potential prognostic genes. Accordingly, we found that 34 MRlncRNAs (m6A-associated lncRNAs) had potential prognostic through the “Survival” package. As shown in Figure 3A, 5 MRlncRNAs had potentially prognostic value (HR > 1, *P* < 0.05), whereas 29 genes showed potential poor value (HR < 1, *P* < 0.05).

**Figure 4:**
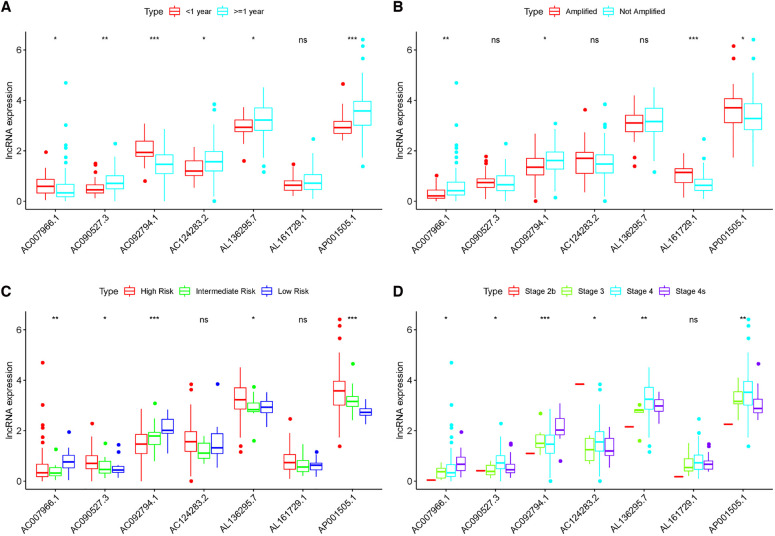
Correlation between the expression level of seven MRlncRNAs in the prediction model and clinical variables. (**A–D**) Most LncRNAs showed significantly different expressions between diverse groups in age (**A**), MYCN (**B**), risk score (**C**), and stage (**D**).

These lncRNAs were taken into consideration to build an MRlncRNA-related prediction model for the prognosis of NBL. To infinitely improve the accuracy of the prediction model, we carried out a LASSO regression analysis *via* the “GLMNet” package (Figures 3B,C). Results showed that the accuracy of the MRlncRNA-related prediction model was the best when 13 MRlncRNAs were selected. Simultaneously, we identified that 7 of 13 MRlncRNAs were independent predictors by multivariate Cox regression (Table 1). The correlation between these lncRNAs and m6A-related genes was further verified (Table 2). We initially established an MRlncRNA-related risk prediction model for NBL. Furthermore, we explored the correlation between these lncRNAs and clinicopathological features. The results showed that most of these lncRNAs, among different subgroups of age, risk score, stage, and amplification status, showed significant differences in expression, indicating that these lncRNAs were significantly correlated with the characteristics of clinical cases. As shown in Figures 4A–D, all lncRNAs except AL161729.1 showed significantly different expressions among diverse groups in age (≤1, >1) and stage subgroups (stage 2b, stage 3, stage 4, stage 4s). In the risk grouping (high risk, intermediate risk, low risk), these lncRNAs except AC0124283.2, and AL161729.1 showed significantly different expressions among diverse groups. Also, similar consequences were observed in the amplification status group (amplified, not amplified).

**Table 1:** Multivariate Cox regression analysis of the MRlncRNAs.

ID	Coef	HR	95% CI	*P* value
AC007966.1	−0.66867	0.512389	0.273306-0.960618	0.037046
AC124283.2	0.370511	1.448475	1.001015-2.095953	0.049374
AL161729.1	0.430911	1.538658	0.914183-2.58971	0.104763
AC092794.1	−0.66539	0.514072	0.322039-0.820616	0.005296
AC090527.3	0.705756	2.025378	1.143209-3.588281	0.015579
AP001505.1	0.507392	1.660954	1.151831-2.395117	0.006591
AL136295.7	0.442733	1.556957	0.952782-2.54425	0.077241

**Table 2:** The correlation between m6A-related genes and targeted lncRNAs.

ID	m6A-related genes	Correlation coefficent	*P* value	Regulation
AC124283.2	RBM15	0.549232	2.19×10^-14^	Postive
AC092794.1	YTHDC2	0.606257	6.19×10^-18^	Postive
AL161729.1	LRPPRC	0.641337	1.70×10^-20^	Postive
AP001505.1	HNRNPC	0.522778	5.90×10^-13^	Postive
AL136295.7	METTL3	0.618525	8.56×10^-19^	Postive
AC007966.1	FTO	0.597777	2.32×10^-17^	Postive
AC090527.3	METTL14	0.50319	5.65×10^-12^	Postive

### The risk prediction model has great predicted performance

**Figure 5:**
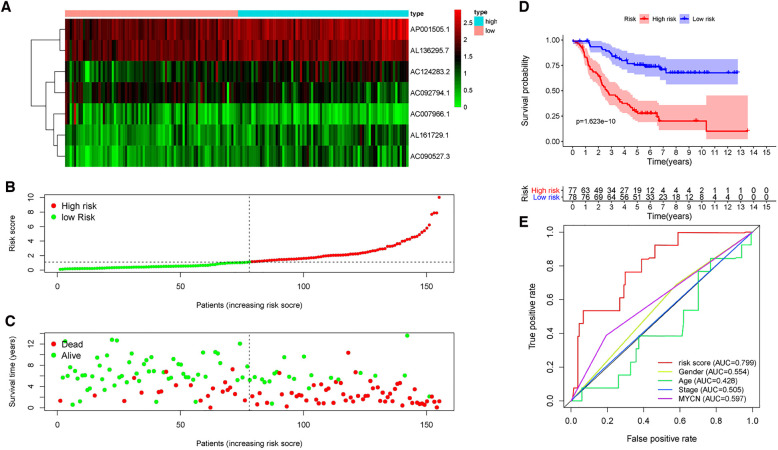
Prognostic value of risk models based on MRlncRNAs. (**A**) The heatmap of differential expression of seven lncRNAs in high- and low-risk groups. (**B**) Risk scores of NBL patients were sorted according to the risk model. (**C**) The scatter plot of the relationship between the risk scores and the survival time in NBL patients. (**D**) Kaplan–Meier overall survival curves showed that patients in the low-risk group exhibited better OS than those in the high-risk group. (**E**) ROC curve analysis showed that the risk score was the best diagnosis predictors compared with other clinicopathological characteristics.

**Figure 6:**
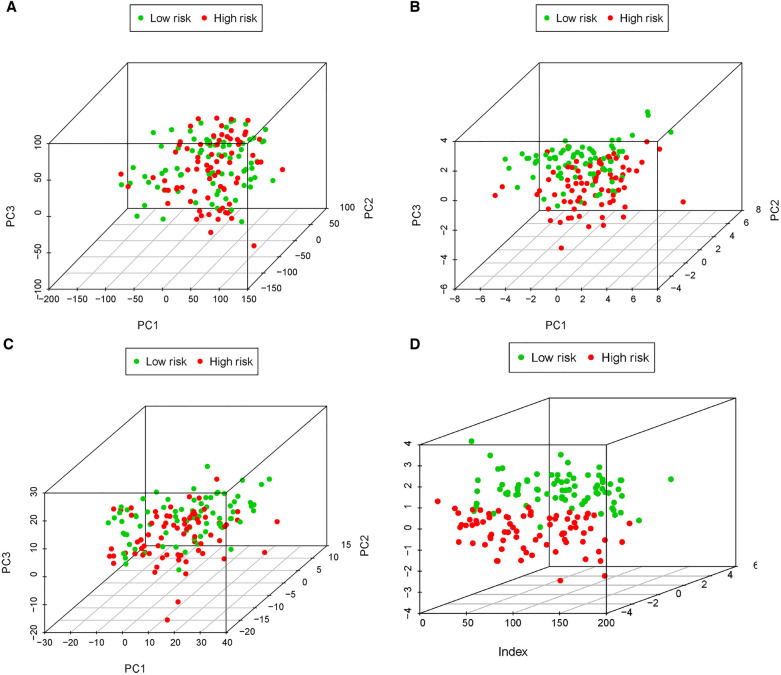
PCA of all expressed genes, m6A genes, 34 MRlncRNAs, and 7 MRlncRNAs in the prediction model. (**A**) Principal components analysis based on whole gene expression profiles. (**B**) Principal components analysis based on m6A-related genes. (**C**) Principal components analysis based on 34 MRlncRNAs. (**D**) Principal components analysis based on 7 MRlncRNAs in the prediction model.

A risk score of each patient was calculated using the formula: risk score = ∑ I Coefficient (MRlncRNAsi) × Expression (MRlncRNAsi). We divided 155 neuroblastoma tissue samples into high- and low-risk groups based on the median value of the risk score. Accordingly, the heatmap hinted at the different expressions of these MRlncRNAs in a diverse risk group (Figure 5A); AC007966.1 and AC092794.1 were highly expressed in the low-risk group, whereas others were highly expressed in the high-risk group. Ranking the patients in the light of risk scores from low to high, we discovered that survival time decreased significantly (Figures 5B,C). Survival analysis using the Kaplan–Meier method based on the risk group showed that patients in the high-risk group had significantly lower survival rates than those in the low-risk group (*P* < 0.001) (Figure 5D). Following that, we explored the prediction accuracy of the risk model by calculating the AUC of the risk score and clinicopathologic features, such as age, gender, stage, and MYCN state, by the ROC curve analysis. The results demonstrated that, relative to other clinicopathological characteristics, the risk score proved to be the optimal diagnosis predictor (Figure 5E). Subsequently, PCA (Figures 6A–D) showed that the samples were notably divided into two risk clusters based on the expression of m6A-related genes (Figure 6B) or seven risk model lncRNAs (Figure 6D).

### The MRlncRNA signature was an independent prognostic indicator

**Figure 7:**
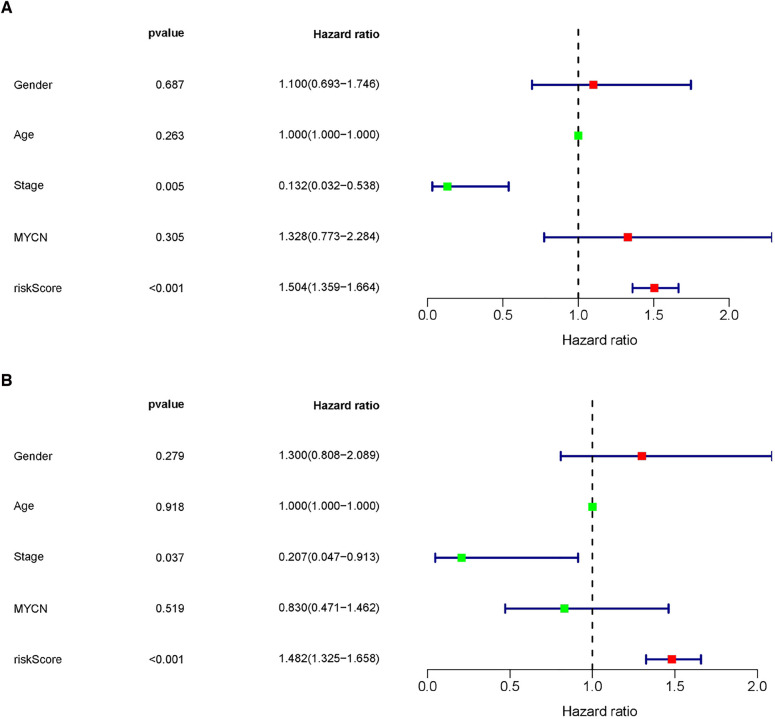
The MRlncRNAs signature was an independent prognostic indicator. Univariate (**A**) and multivariate (**B**) Cox regression analysis of the MRlncRNAs signature with clinicopathological features (*P* < 0.05).

Following considering the clinicopathological features, to determine the influence of clinicopathological features regarding the risk score based on the predictive model, univariate and multivariate regression analyses were used once more to assess the independence of predictive function concerning the risk score (Figures 7A,B). The results revealed that undergoing adjusting other clinicopathologic features, such as age, sex, stage, and MYCN amplification by multivariate Cox regression analysis, risk score remained an independent predictor [HR = 1.482, 95% CI (1.325–1.658); *P* < 0.001].

**Figure 8:**
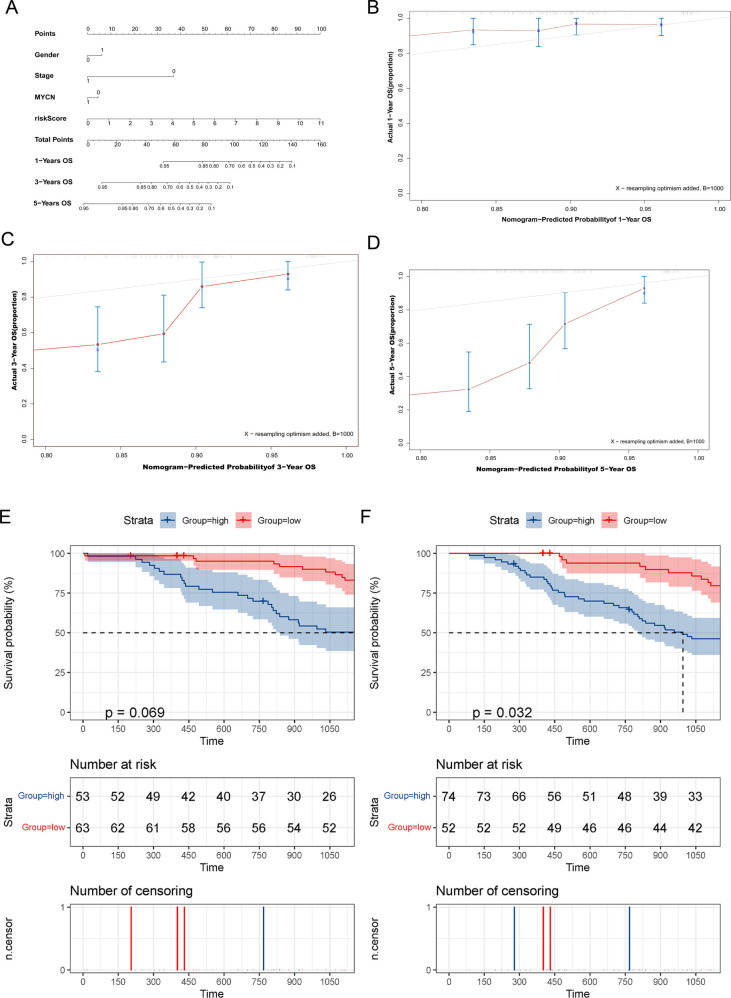
The MRlncRNAs possessed potential clinical value. (**A**) A nomogram of patients’ clinical information, risk score, and OS. (**B–D**) Calibration plots for assessing agreement between the predicted and the actual OS at 1, 3, and 5 years. The survival curves of the MRlncRNAs signature stratified by the nonamplified MYCN group (**E**) and higher-age group (**F**).

Furthermore, the nomogram showed that the risk score contributed the most to OS (Figure 8A). It was also discovered that the 3- and 5-year survival rates of patients were very low. Subsequently, we tested the prediction accuracy of the risk prediction model by the calibration curve, the results of which showed that the prediction accuracy of 1-year survival rates was relatively high (Figure 8B), while the prediction accuracy of 3- and 5-year survival rates were relatively poor (Figures 8C,D), which may be due to the small number of cases.

**Figure 9:**
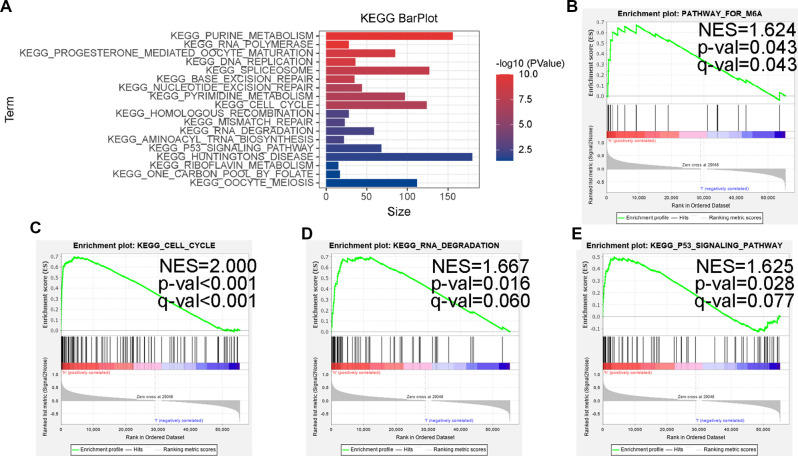
Some cancer-related hallmarks and immunologic features regulated through the MRlncRNAs signature. (**A**) KEGG pathway enrichment analysis of the MRlncRNAs signature. The top enrichment KEGG pathways including FOR-m6A (**B**), CELL-CYCLE (**C**), RNA-DEGRADATION (**D**), and P53-SIGNALING-PATHWAY (**E**).

For conducting internal detection of the risk model, we classified patients according to their risk scores and tested the survival rates of different subgroups. As shown in Figures 8E,F, the survival rates of the high-risk group were significantly lower than that of the low-risk group within the nonamplified MYCN group and higher-age group, whereas the significantly different surgical rates have not been observed in other subgroups, which may be due to the small number of patient cases. Subsequently, we performed a KEGG pathway enrichment analysis of prognosis-related MRlncRNAs. As shown in Figures 9A–E, the concentrations of pathway enrichment were mainly in “FOR_m6A,” “CELL_CYCLE,” “RNA_degradation,” and “P53_signaling,” indicating that this lncRNA was related to m6A and RNA_degradation, which was consistent with our study. Furthermore, these MRlncRNAs may also affect the progression of NBL by the cell cycle and P53 signal pathway.

## Discussion

NBL is one of the most aggressive and poorly proposed solid tumors in children (22). As currently validated predictive tools, the International Neuroblastoma Staging System (INSS) stage, International Neuroblastoma Risk Group Staging System (INRGSS), and Children's Oncology Group (COG) risk stratification have been used to reflect and predict the progression and prognosis of NBL (23, 24). Currently, risk assessment systems for tumor patients are converging toward an integrated assessment of clinical, histopathological, and genetic prognostic markers (25–27). The COG pediatric NBL risk prediction model integrates clinical and histopathological factors while incorporating the MYCN gene. MYCN is the only biological marker for risk assessment. The results of clinical and molecular studies suggest that current risk assessment systems remain inadequate (28, 29), leading to over- or undertreatment of a small proportion of patients. In the clinical setting, new therapeutic prognostic markers are urgently required to improve the prognosis of NBL patients, design more effective treatments, and to better target the heterogeneity of NBL. In recent years, several studies have proposed that molecular markers, such as copy number alterations, gene expression classifiers, and somatic mutation patterns, can accurately reflect tumor biology. Furthermore, it has been shown that epigenetic alterations are closely associated with the development of NBL (30–33). Compounds targeting epigenetically modified proteins are increasingly evaluated as anticancer therapeutic agents, for m6A modifications involved in the development of NBL (34, 35). One study suggested that risk models constructed from m6A-related mRNAs may effectively predict the prognosis of children with NBL (34). Nonetheless, as of now, no study has reported on the association of m6A-related lncRNAs with NBL prognosis.

Through a systematic analysis of 21 widely reported m6A-related genes, lncRNA expression, and clinical features in 155 NBL tissues, m6A methylation regulators are the most abundant form of methylation modification in lncRNAs and play an important role in the prognosis of NBL. Traditional stratified treatment strategies have made progress in improving the overall survival of NBL patients, while the result of patients with highly aggressive NBL remains unsatisfactory (29, 36). In the past few decades, there have been numerous in-depth studies on DNA and mRNA methylation, but few studies on lncRNA methylation. By analyzing transcriptomic data, differential expression of m6A-associated lncRNAs was found to be significantly associated with NBL malignancies. These seven genes constituted independent predictors of prognosis, and the predictive value of the risk model was verified in the independent dataset target. To further improve the prognostic accuracy, the inclusion of several gene expression-based risk estimation scores, integrating several prognostic determinants, such as age, tumor stage, and MYCN oncogene amplification, may be a useful addition to the INSS stage and COG risk stratification. As a new predictive method, molecular prognostic markers that can be quantified by standardized detection procedures change as the tumor progresses, thereby dynamically reflecting the prognosis of patients. Based on the model principal component analysis, the predictive model readily distinguishes between two major subgroups, including patients with good outcomes and those at high risk of dying from the disease. All seven genes included in the model were reported for the first time in NBL. These molecular prognostic markers may also be potentially valuable in early diagnosis, thus providing a new research direction to study the molecular mechanisms underlying the development of NBL. Nomogram is widely used in clinical oncology for evaluation due to the fact that it integrates molecular and clinicopathological parameters (37–40). The probability of each event can be relatively simply calculated and visualized. Nomogram combines the characteristics of seven genes and four clinicopathological parameters. The graphical scoring system is easy to understand and assists in personalized treatment and medical decisions.

In terms of molecular mechanism, we find that the high-risk group was closely associated with the characteristics of malignancy, including P53 signaling pathway, cell cycle, and DNA repair, by enrichment analysis of relevant lncRNA GSEA, which is in line with the current extensive findings on the regulation of lncRNA involvement in the cell cycle in NBL (41–43). Notably, the high-risk group was enriched in the m6A database.

However, the present study still bears several limitations. First, the conclusions are based on the analysis and summary of the TARGET database with fewer numbers; thus, due to the lack of a similar data set, it becomes difficult to verify the validity of this model. Second, the interaction between m6A regulators and prognostic lncRNAs needs future confirmation by assay data. Therefore, future studies with a larger sample size and basic experiments are needed to further confirm our conclusions.

In conclusion, the study constructed a new model of NBL prognosis based on m6A-associated lncRNAs. Accordingly, the prognostic genes identified in this model could serve as novel biomarkers of prognosis in NBL patients, enabling clinicians to adopt individualized treatment plans. In addition, these findings guide basic medical research on m6A methylation in neuroblastoma.

## Data Availability

The original contributions presented in the study are included in the article/**Supplementary Material**, further inquiries can be directed to the corresponding author.
