# Supplementary material for: Characterization of m6A-related lncRNA signature in neuroblastoma
Source: Front Pediatr. 2022 Oct 17;10:927885. doi: 10.3389/fped.2022.927885 (PMC9618704; doi:10.3389/fped.2022.927885)
Supplement: Supplementary file 2 [file DataSheet1.pdf]

All raw data are available in the Jianguoyun
website (https://www.jianguoyun.com/). The raw data were downloaded
at: https://www.jianguoyun.com/d/home#/sandbox/15ddfac/3e02d0153abe6074/%2F/
